# Peptidomimetics in cancer targeting

**DOI:** 10.1186/s10020-022-00577-3

**Published:** 2022-12-07

**Authors:** Mohammad Mahmoudi Gomari, Shadi Abkhiz, Taha Ghantab Pour, Ehsan Lotfi, Neda Rostami, Fatemeh Nafe Monfared, Babak Ghobari, Mona Mosavi, Behruz Alipour, Nikolay V. Dokholyan

**Affiliations:** 1 Department of Medical Biotechnology, Faculty of Allied Medicine, Iran University of Medical Sciences, Tehran, Iran; 2 Department of Anatomy, School of Medicine, Iran University of Medical Sciences, Tehran, Iran; 3 Department of Chemical Engineering, Faculty of Engineering, Arak University, Arak, Iran; 4 Department of Virology, School of Public Health, Tehran University of Medical Sciences, Tehran, Iran; 5 Department of Biology, Faculty of Natural Sciences, University of Tabriz, Tabriz, Iran; 6 Medical Biotechnology Department, School of Advanced Technologies in Medicine, Tehran University of Medical Sciences, Tehran, Iran; 7 Department of Pharmacology, Penn State College of Medicine, Hershey, PA USA; 8 Department of Biochemistry & Molecular Biology, Penn State College of Medicine, Hershey, PA USA

**Keywords:** Peptidomimetic, Angiogenesis, Nanoparticles, Drug resistance, Metastasis, Apoptosis

## Abstract

The low efficiency of treatment strategies is one of the main obstacles to developing cancer inhibitors. Up to now, various classes of therapeutics have been developed to inhibit cancer progression. Peptides due to their small size and easy production compared to proteins are highly regarded in designing cancer vaccines and oncogenic pathway inhibitors. Although peptides seem to be a suitable therapeutic option, their short lifespan, instability, and low binding affinity for their target have not been widely applicable against malignant tumors. Given the peptides’ disadvantages, a new class of agents called peptidomimetic has been introduced. With advances in physical chemistry and biochemistry, as well as increased knowledge about biomolecule structures, it is now possible to chemically modify peptides to develop efficient peptidomimetics. In recent years, numerous studies have been performed to the evaluation of the effectiveness of peptidomimetics in inhibiting metastasis, angiogenesis, and cancerous cell growth. Here, we offer a comprehensive review of designed peptidomimetics to diagnose and treat cancer.

## Background

Despite significant advances in molecular biology and a fundamental understanding of cancer biology over the past few decades (Aghamiri et al. 2019; Rostami and Davarnejad 2021), cancer remains the second leading cause of global mortality (Marques et al. 2020; Rahim et al. 2021; Gomari et al. 2021). As conventional cancer treatments, such as chemotherapy, can cause serious side effects in cancer patients, alternative therapies can be helpful (Mahmoudi Gomari et al. 2021). Among them, bioactive peptides derived from natural sources have been evaluated because of their potential to treat chronic diseases such as cancer (Quintal-Bojórquez and Segura-Campos 2021; Mahmoudi Gomari et al. 2020; Hao et al. 2008). However, the synthesis and purification of therapeutics peptides are not straightforward because of low yields, chemical instability, hydrolysis, short half-life, and complex transfer methodologies. Moreover, peptides are typically disordered and prone to proteolytic degradation, resulting in low binding affinities. To overcome these limitations, peptides containing modified amino acids or chemical modifications can be used to produce peptidomimetics (Qvit et al. 2017; Evans et al. 2020).

The term “peptidomimetics” describes the modification of peptide sequences with improved biological properties and the replacement of the peptide backbone with a molecular scaffold (Kuppusamy et al. 2019). They are designed to be metabolically stable, bioavailable, and highly selective (Table 1).

**Table 1:** Comparing peptidomimetics attributes with native peptides

Characteristics	Peptides	Peptidomimetics	References
Stability	Limited stability toward proteolysis	Higher stability toward proteolysis	Trabocchi (2019), Li Petri et al. (2022), Trabocchi and Guarna (2014)
Interactions/ Specificity	✓ Low selectivity due to high backbone flexibility ✓ Interaction with multiple targets	✓ Higher receptor affinity and selectivity ✓ Pharmacophore-based target selection	
Transport system	Rapid excretion, poor cell permeability, lacks specific transport systems due to its relatively high molecular mass	✓ Transports from Peptide transporter 2 (PEPT2) which is a low-capacity/high-affinity proton-coupled cotransporter of diverse di- and tripeptides ✓ The di/tripeptide transporter in the small intestine absorbs some of them	
Activity	Limited activity	Higher activity	Kim et al. (2015)
Duration of action	Shorter duration of action	longer duration of action	

Producing peptidomimetics can be accomplished in several ways, including the following: (i) manipulating native peptide backbones, (ii) coupling unnatural amino acids derived from modifications, (iii) replacing amino groups with oxygen or sulfur by isosteric substitution (Gomes Von Borowski et al. 2018; Wang et al. 2016). Structure, sequence, function, and protein binding site characteristics are all factors that influence the design strategy (Lenci and Trabocchi 2020). In addition, the secondary structure of the amino acid sequence is essential for interaction with the target, so it must be considered in peptidomimetics design (Mizuno et al. 2017). Peptidomimetic compounds are used in a wide range of cancer-related conditions from diagnosis to treatment. As well as apoptotic regulators, membrane receptors, small GTPases, and transcriptional regulators, their application has been tested on various protein model systems (Mabonga and Kappo 2020). Some of the anti-cancerous peptidomimetics that have entered clinical trials are listed in Table 2.

**Table 2:** Some key features of anticancer peptidomimetics that have entered the clinical studies

Name	Target	Indication	Clinical status/Trial ID	Refs.
68Ga-NODAGA-THERANOST™	αvβ3 integrin	✓ Non-small-cell lung cancer ✓ Breast cancer	✓ Phase 2 ✓ NCT04480619	Baum et al. (2015), Kim et al. (2012)
64Cu-LLP2A	α4β1 integrin	Multiple myeloma (MM)	✓ Early Phase 1 ✓ NCT03804424	Beaino and Anderson (2014), Walker et al. (2016), Laforest et al. (2022)
FAP-2286	Fibroblast Activation Protein (FAP)	Solid tumors	✓ Phase 1 ✓ NCT04621435	Zboralski et al. (2022)
Cilengitide	αvβ3, αvβ5, α5β1 integrins	✓ Malignant glioblastoma (GBM) ✓ Malignant primary brain tumors	✓ Phase 3 ✓ NCT00689221	Mas-Moruno et al. (2010), Dechantsreiter et al. (1999), Stupp et al. (2014)
GDC-0152	ANGPTL2	Solid tumors	✓ Phase 1 ✓ NCT00689221	Shin et al. (2013), Yang et al. (2015)
KX2-391	Src kinase inhibitor	Solid tumors	✓ Phase 2 ✓ NCT02838628	Kempers et al. (2020)
Cyclic RGD	VEGF related pathways	Colon and pancreatic malignancies	✓ Phase 2 ✓ NCT05518071	Valk et al. (2020)
XK469	DNA topoisomerase II (topo II) inhibition	Liver tumors	✓ Phase 1 ✓ NCT00028548	Xia et al. (2016)

Protein–protein interactions (PPIs) are involved in most cellular processes and affect biological functions. Modulating PPIs is regarded as a promising treatment strategy and peptidomimetics appear to be an essential tool for targeting PPI in future drug discovery (Dagliyan et al. 2011; Allen et al. 2016). However, peptidomimetic chemistry is not only about designing PPI inhibitors, but it has been a long-standing goal in chemical sciences (Pelay-Gimeno et al. 2015). This study aims to summarize peptidomimetics as a potent alternative for peptides in cancer targeting.

## Peptidomimetics classification

Peptidomimetics can be classified into two different forms. Traditionally the first classification is based on structural/functional properties and divided into type I, type II, and III. Structural, functional, and functional-structural mimetics are related to type I, type II, and type III, respectively. Based on the literature, group replacement assisted binding (GRAB)-peptidomimetic is another type of introduction known as type IV (Ripka and Rich 1998; Tomasella et al. 2021). Structural mimetics are short peptides that mimic the local topography of the amide bond. In contrast, functional mimetics may not necessarily mimic the structure of the parent peptide, though they identify the biological target. A structural/functional mimetic is a non-peptide template that shows topological similarity with the parent peptide but does not exhibit a direct atom-by-atom analogy (Pelay-Gimeno et al. 2015; Floris and Moro 2012). The last one, type IV or non-peptide mimetics, is structurally similar to type I peptidomimetics but binds an enzyme form that cannot be accessed by type I (Kharb et al. 2011).

**Fig. 1:**
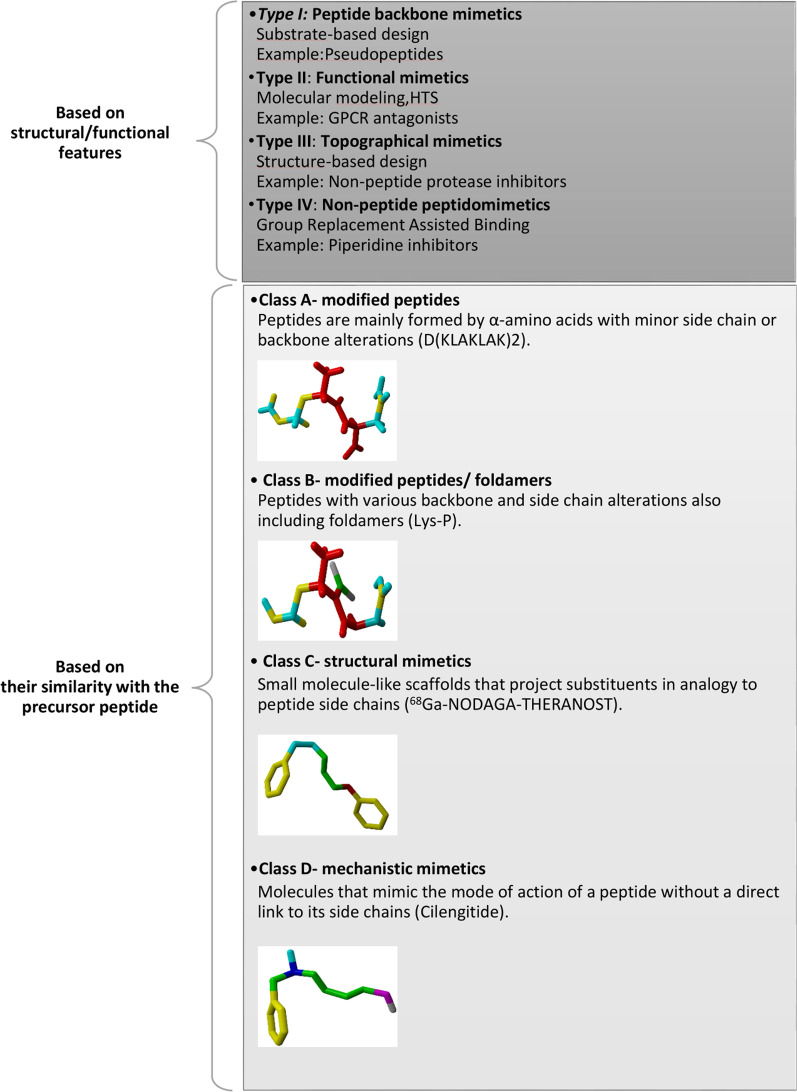
Peptidomimetics classification

Recently, a new classification of peptidomimetics was proposed based on their similarity to natural peptide precursors (Abdildinova et al. 2021). As a result, four classes are identified as A–D, the most similar being A while the least similar is D (Fig. 1). Class A peptidomimetics, which are very similar to type I, are structures that mainly consist of parental peptide amino acid sequences. In class B mimics, different amino acids are substituted for their natural counterparts, desired molecule features are defined, and major chemical alterations are made to the backbones of the molecules. Peptidomimetics belonging to this class are similar to types II classical peptidomimetics. Class C mimics, which are related to type III peptidomimetics, consist of highly modified structures with small molecule properties that replace the peptide backbone completely. Finally, mimetics of Class D resemble bioactive peptides without being directly linked to their side chains. Depending on how far it is abstracted from the parent peptide, this class might parallel the traditional type II or type III peptidomimetics (Trabocchi 2019; Lenci and Trabocchi 2020).

## Peptidomimetics facilitated cancer diagnosis

Cancer cells have cell surface receptors that are up-regulated and overexpressed in the plasma membrane. These receptors are absent or present at a much lower level in normal cells, allowing them to be used for cancer-targeted detection (Shah et al. 2020). Peptidomimetics may be able to act on diagnostic areas by molecular imaging. There has been rapid progress in the field of molecular imaging due to its potential for improving diagnosis, staging, and treatment strategies for cancer. Diagnostic imaging techniques based on molecular imaging are one of the most beneficial, non-invasive tools available for visualizing, describing, and measuring biological processes on a cellular and molecular level (Okarvi 2019). Magnetic resonance imaging (MRI), positron emission tomography (PET), computed tomography (CT), and single-photon emission computed tomography (SPECT) are the most common imaging techniques used to diagnose cancer at different stages. Biochemical markers such as proteins, DNAs, mRNAs, and microRNAs can be used as new diagnostic tools along with imaging techniques. Although imaging techniques are very efficient, they are not without limitations, such as their high price (Jafari et al. 2018).

One promising approach to identifying molecular markers is to use imaging probes conjugated to specific ligands that recognize overexpressed receptors in angiogenic tumors; integrin is an example of the target (Cai and Chen 2008). The diagnostic applications of peptidomimetics are related to several synthetic peptides and peptidomimetics that are potent ligands and antagonists of integrins and inhibit integrin-mediated tumor-stimulating biological mechanisms. There are numerous integrins associated with poor prognosis, including αvβ3, αvβ5, α5β1, α6β4, α4β1, αvβ6 and αvβ8; most relevant studies focus on αvβ3. For this integrin, there is growing interest in peptidomimetics that mimic the guanidine and carboxylate pharmacophores present in RGD (Arg-Gly-Asp) peptides (Kim et al. 2015; Nieberler et al. 2017). Integrin-based imaging for αvβ3 can be classified into two distinct groups, including radionuclide and nanoparticle-base. 18F-Galacto-RGD, 99mTc-NC100692, and 18F-fluciclatide are three radiotracers that have been developed for PET and SPECT in the radionuclide categories. The first approach to image expression of αvβ3 with nanoparticles was paramagnetic liposomes containing Gd3^+^. There have also been various types of nanoparticles developed for cancer imaging, including quantum dots (QDs), carbon nanotubes, and gold nanoparticles (Danhier et al. 2012). In a study by Kim et al. in 2012, the authors reported αvβ3 integrin is over-expressed in various human tumors. According to this research, the peptidomimetic αvβ3 integrin antagonist (IAC), easily synthesized and controlled, helps tumor PET and SPECT imaging.

Some researchers have also reported a novel class of RGD peptidomimetics containing a bifunctional diketopiperazine (DKP) scaffold, which has shown a low nanomolar affinity for both integrins α_v_β_3_ and α_v_β_5_ (Marchini et al. 2012; Ressurreição et al. 2009). Several linear and multimeric peptidomimetics including the RGD motif have been proposed for therapeutic applications or molecular imaging of integrin expression based on previous studies (Goodman and Picard 2012; Schottelius et al. 2009; Meyer et al. 2006; Thumshirn et al. 2003). Cilengitide has denoted significant inhibition of tumor angiogenesis in many preclinical studies. It is undergoing clinical testing for several cancer types (Mas-Moruno et al. 2010; Dechantsreiter et al. 1999). To develop SPECT and PET tracers, galactose- or glucose-based sugar amino acids were conjugated to cyclic pentapeptides (Haubner et al. 2001, 2004; Gruner et al. 2002). Recent studies have documented that the role of integrin α_v_β_3_ is complicated, so interpreting imaging data has often become complex in terms of both clinical importance and biological meaning (Robinson and Hodivala-Dilke 2011; Avraamides et al. 2008).

Some other reports indicate the synthesis of 68Ga-NODAGA-THERANOST and its clinical evaluation for integrating integrin receptor imaging demonstrate the potential for identifying cancer patients who could benefit early in their healing process using this peptidomimetic for imaging. Based on preliminary evidence, this radioligand could be helpful for early tumor detection and non-invasive monitoring of tumor metastasis in a range of conditions. This radiolabeled peptidomimetic was confirmed to work in patients with non-small cell lung cancer and breast cancer in the first-in-human study (Baum et al. 2015). Furthermore, targeting other integrin subtypes, such as α5β1, is of great interest due to its critical role in metastatic cell colonization, the resistance of tumor cells to chemotherapy and ionizing radiation, and tumor invasion. Consequently, the design and synthesis of antagonists that inhibit specific sites in RGD integrin are of great interest, both for the development of treatments of choice and for selective molecular imaging of this integrin subtype (D’Alessandria et al. 2015).

Furthermore, in another study by Walker et al. the peptidomimetic, LLP2A has been introduced as a specific, high-affinity ligand for α4β1 integrin receptors. They report the first 18F-labeling of the α4β1 integrin-specific LLP2A peptidomimetic, which was the first step in the development of an expanding library of 18F-R-BF3 LLP2A radiotracers for PET imaging (Walker et al. 2016). In the case of LLP2A, 64Cu-LLP2A has been suggested to allow accurate molecular imaging of multiple myeloma (MM) lesions, which will significantly impact the diagnosis of the disease at its early stages and, over time, on the diagnosis and selection of treatment in these patients (Beaino and Anderson 2014). FAP-2286 is another study for diagnostic peptidomimetics, which binds to fibroblast activation protein (FAP) to evaluate the uptake, retention, and ability to detect metastatic disease of radiotracers in a variety of solid tumors (Zboralski et al. 2022). In general, molecular imaging is one promising potential factor for cancer diagnosis with peptidomimetics. Another method for cancer diagnosis by peptidomimetics is to target PPI's (Mabonga and Kappo 2020). This goal is achieved by mimicking the peptide-binding epitopes in their bioactive composition, but few studies have been done in this area. Besides integrins, other biomolecules have also been targeted for cancer diagnosis using peptidomimetics. Human breast cancer and cervical carcinomas are associated with increased expression of CRIP1, a member of the LIM/double zinc finger protein family. Hao and his colleagues designed a cyclic peptide called A1M (CLDGGGKGC) as a potent ligand for CRIP1 using phage display and computational approaches. Molecular imaging of malignant tissues can be achieved using A1M which binds to CRIP1 at micromolar concentrations (Hao et al. 2008).

## The ability of peptidomimetics on angiogenesis inhibition

Angiogenesis has a critical function in the growth, nutrition, and invasion of malignant cells. Consequently, targeting this biological process plays an important role in inhibiting cancer and increasing the chances of patients’ survival (Nishida et al. 2006). Interferon-γ (IFNγ) is a cytokine primarily produced by natural killer (NK) and T cells. A peptidomimetic derived from IFNγ (known as mimγ) which lacks the amino-terminal IFNγR-binding sequence revealed therapeutic potency (Bansal et al. 2014a, b). Besides, Fibroferon which was designed by fusion of mimγ to the bicyclic platelet-derived growth factor receptor-beta recognizing peptide has a great ability for targeting PDGFβR expression (Dijk et al. 2015). Poosti et al. (2016) showed that Fibroferon can diminish angiogenesis and/or lymphangiogenesis by reducing the number of podoplanin^+^ lymphatic capillaries and CD31^+^ peritubular capillaries compared to targeted full-length IFNγ in the mice with unilateral ureteral obstruction.

The Src pathway is another target to inhibit angiogenesis in cancerous tissue (Hanahan and Weinberg 2000, 2011). It was demonstrated Src kinase has been associated with breast cancer proliferation, angiogenesis, cell motility, migration/invasion, and metastasis (Irby and Yeatman 2000). The KX-01 (clinical reference, KX2-391) was designed as a “First-in-Class” peptidomimetic Src kinase inhibitor that attaches to the peptide substrate pocket and inhibits kinase activity and downstream components of Src-related pathways (Sakamoto et al. 2001; Schlessinger 2000). Anbalagan et al. (2012) showed that KX-01 significantly reduced the microvessel density (MVD) of MDA-MB-231 and MDA-MB-157 (human breast cancer cell lines) tumor xenografts.

Targeted proapoptotic peptides are short peptides composed of two functional domains: a blood vessel homing motif and a programmed cell death-inducing sequence (Smolarczyk et al. 2006). Lahdenranta et al. (2007) showed that a single injection of the RGD-4C-peptide fused to the proapoptotic moiety [RGD-4C-GG-D(KLAKLAK)2] significantly induces endothelial cell apoptosis and a subsequent reduction in retinal neovascularization.

**Fig. 2:**
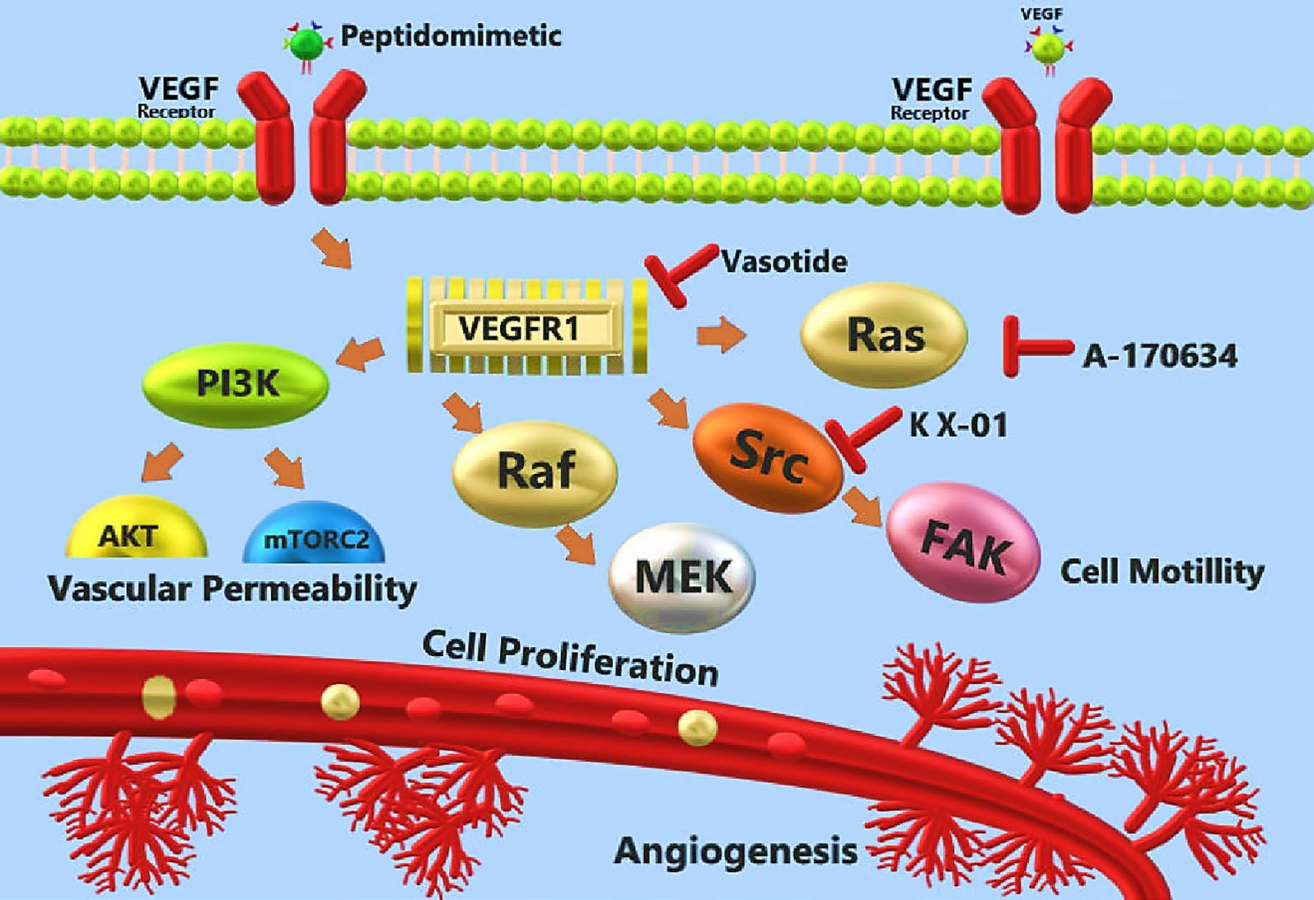
Implementation of peptidomimetics against tumor angiogenesis

So far, many efforts have been made to control of VEGF pathway, which plays a key role in the angiogenesis of malignant tumors. Vasotide (_D_(Cys-Leu-Pro-Arg-Cys)) is a potent peptidomimetic that was designed for angiogenesis targeting. Vasotide is a VEGF receptor-targeted prototype peptidomimetic that binds effectively to NRP-1 and VEGFR-1 (Wilkinson-Berka and Deliyanti 2016). Richard et al. (Sidman et al. 2015) have shown that the Vasotide can reduce pathological angiogenesis in two preclinical murine models and one nonhuman primate model of human retinal diseases. A-170634 is another VEGF inhibitor peptidomimetic. This therapeutic is a novel CAAX peptidomimetic that inhibits Ras oncogene and blocks anchorage-dependent and independent growth of HCT116 K-*Ras* mutated cells by inhibiting farnesyltransferase activity over the closely related geranylgeranyltransferase I. Gu et al. (1999) showed that A-170634 decreased human umbilical vein endothelial cells (HUVEC) capillary structure formation, VEGF secretion from HCT116 colon cancer cells, and HUVEC growth stimulating activity in a dose-dependent manner by inhibition of Ras processing that participates in the tumor angiogenesis (Fig. 2).

Cyclic RGD peptidomimetics carrying a bifunctional diketopiperazine scaffold are a novel category of high-affinity ligands for the integrins α_V_β_3_ and α_V_β_5_. Fanelli et al. (2014) showed that cyclic RGD peptidomimetic *cyclo*[DKP-RGD] 1 effectively inhibited angiogenesis induced by the growth factors VEGF, EGF, IGF-I, and FGF2. As well as this peptidomimetic antagonizes the pathological angiogenesis activity of pro-inflammatory chemokine IL-8 in HUVEC.

## Apoptosis triggering by peptidomimetics

Apoptosis (programmed cell death) is a regulated biological process known as “cell suicide.” As a carefully regulated event, apoptosis results in cell shrinkage and fragmentation. Apoptosis is done in two ways; physiological which occurs as part of a biological function or normal development of tissue, and pathological as part of a disease. In a pathological scenario, there are two major cell-intrinsic pathways for inducing apoptosis, one that begins with the ligation of cell surface death receptors and another that involves the mitochondrial release of cytochrome *c* (Elmore 2007). Therapeutic interventions can affect apoptosis in either of these latter two types. Peptidomimetics illustrate a novel type of agent that interacts with the peptide substrate pocket of proteins. As said previously, a peptidomimetic is an advantageous method to enhance the drug-like properties of peptide-based inhibitors by improving their stability and biological activity (Levesque et al. 2015). Another well-known pathway in apoptosis is the P53 signaling pathway. The interaction of p53 as a tumor suppressor with MDM2 is a critical regulator of the intrinsic pathway of apoptosis (Hu et al. 2007). Due to the importance of water-soil interaction, many efforts have been made to control the formation of their complex. Compounds such as ^D^PMI-α ^D^PMIs, M06, sMTide-02/02A, ATSP-7041 (as MDM2 inhibitors), p53p-Ant, Peptide 46, and ReACp53 which target p53/MDM2 circuitry are some peptidomimetics designed for this purpose (Teveroni et al. 2016).

PACE4 is a member of the proprotein convertases family of enzymes that is used as a therapeutic target in several cancers (Fugère and Day 2005). Multi-Leu (ML) peptide is a PACE4 inhibitor with potent anti-cancer effects (Levesque et al. 2012). Although the ML-peptide (Ac-LLLLRVKR-NH2) shows great promise as a lead compound, its high clearance rate significantly affects its beneficial pharmacological effects (Levesque et al. 2015). One potential solution to this problem is to maintain the basic structure of the molecule while protecting it from proteases using peptidomimetic approaches (Kwiatkowska et al. 2014). Using a decarboxylated amidinobenzylamide (Amba) arginine mimetic as a replacement of C-terminal Arg, the stability of the ML-peptide inhibitor significantly increased. The N-terminal segment of ML-peptide inhibitor is less problematic and its protection from aminopeptidases was accomplished by the substitution of the N-terminal leucine with a d-leucine isomer. Levesque et al. (2015) combined these two modifications (d-Leu^P8^ and Amba^P1^) to evaluate the inhibitory potency of the ML-peptide analog in DU145 and LNCaP prostate cancer cell lines. The peptidomimetic Ac-[DLeu]LLLRVK-Amba is a low nanomolar PACE4 inhibitor in vitro with a fourfold increase in potency when compared to ML peptide. The cell-cycle analysis performed on LNCaP cells treated with Ac-[Dleu]LLLRVK-Amba peptide reveals a dose–response G_0_/G_1_ cell cycle arrest along with increased apoptotic events (Levesque et al. 2015; Kwiatkowska et al. 2019).

A Bim-derived BH3 mimetic drug was recently synthesized by replacing specific residues of the wt Bim-BH3 sequence with natural and non-natural amino acids to increase the affinity for Bcl-X_L_ (Ponassi et al. 2008). The peptide was further modified to enhance its stability in serum and boost its capacity to penetrate cell membranes. The result was named 072RB. Ghiotto et al. (2009) demonstrate that 072RB peptidomimetic induces significant apoptosis of B-CLL cells subsequent to Bcl-XL and Mcl-1 downregulation. KX-01 was another peptidomimetic capable of inducing apoptosis. In vitro analysis showed that KX-01 extremely induced apoptosis in MDA-MB-468 cells (human breast cancer cell line) at 25 nmol/L, whereas dasatinib (specific Src/tyrosine kinase inhibitor) did not induce apoptosis at a × 10 higher concentration (Lahdenranta et al. 2007; Anbalagan et al. 2017).

**Fig. 3:**
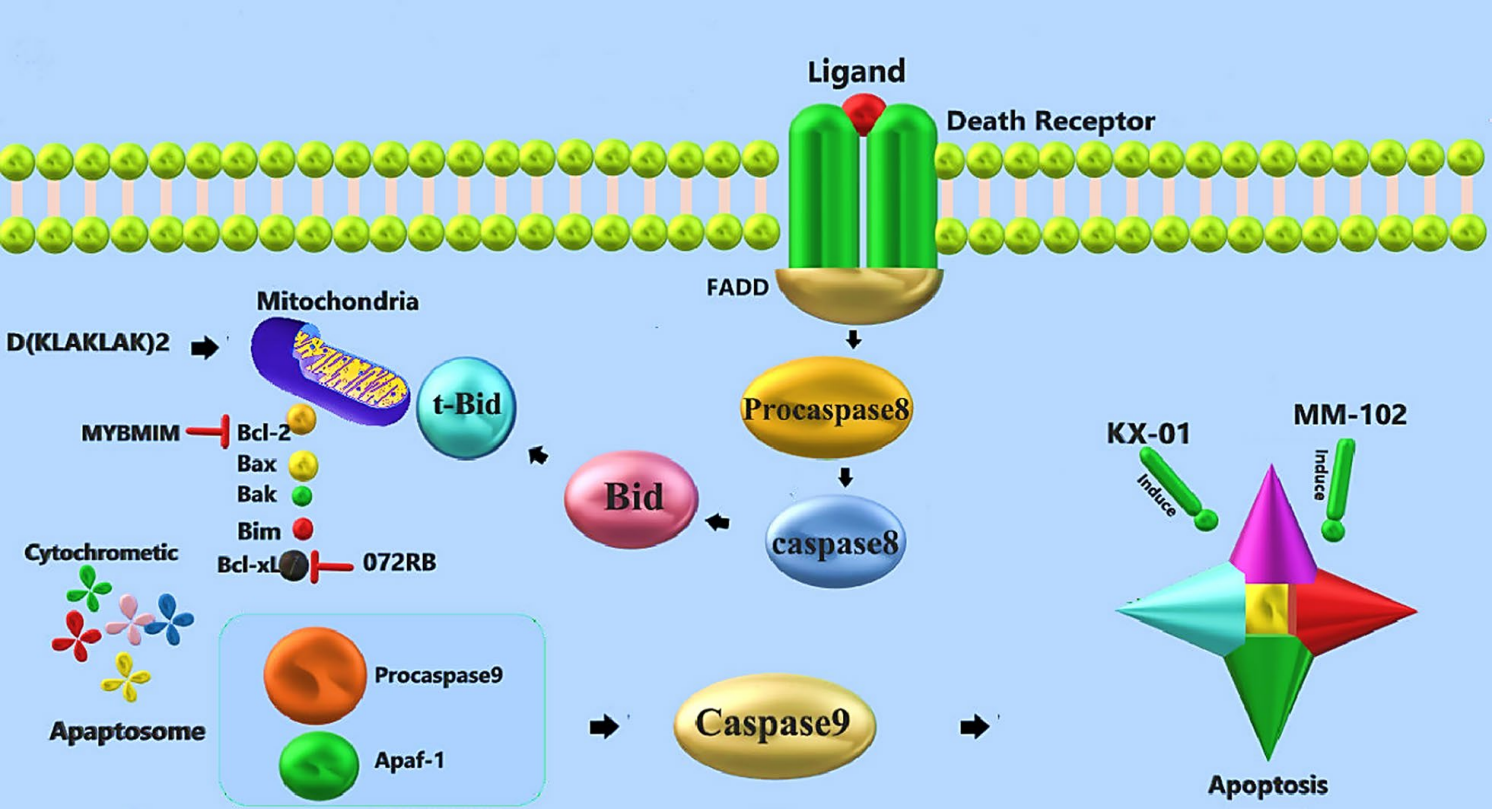
Triggering apoptosis pathway using peptidomimetics

Some designed peptidomimetics like all-D-enantiomer can induce the mechanism of plasma membrane degradation. While others like D(KLAKLAK)2 enter the cell and trigger mitochondrial membrane depolarization and swelling resulting in apoptosis. D(KLAKLAK)2 peptidomimetics can increase caspase-like enzymatic activity Mucorales cells (Barbu et al. 2013). Sometimes peptidomimetics work by inhibiting the BCL2 expression. MYBMIM is a peptidomimetic inhibitor that interferes with the assembly of the MYB:CBP/P300 complex and rapidly accumulates in the nuclei of AML cells. Ramaswamy et al. (2018) showed that MYBMIM downregulated the MYB-bound BCL2 enhancer, leading to the downregulation of BCL2 expression and apoptosis of leukemia cells (Fig. 3). MLL1/WDR5 targeting is another strategy for triggering cell apoptosis. MLL1/WDR5 protein–protein association is crucial for MLL1 enzymatic activity. MM-102 is a potent WDR5/MLL interaction inhibitor. This peptidomimetic has a high-affinity binding to WDR5. Karatas et al. (2013) demonstrated that MM-102 can inhibit cell growth and induce apoptosis in leukemia cells harboring MLL1 fusion proteins.

## Developed peptidomimetics for cancer proliferation targeting

Peptidomimetics represent substantial antiproliferative ability against tumor cells in vitro and in vivo investigations. They target the proliferation of cells in different ways. For instance, SRC2-SP3, PERM1, and ER-1b are peptidomimetics that target proliferation by targeting estrogen receptor alfa (ER-α) (Qin et al. 2018). Peptoids are a class of peptidomimetics in which the side chains are appended to the amide nitrogen atoms, engendering proteolytic stability and improved cellular permeability (Miller et al. 1995). The sequence-specific assembly of peptoids enables precise tuning of ligand valency and spacing to enhance affinity and specificity for corresponding biomolecular targets (Childs-Disney et al. 2011). Levine et al. (2012) indicated that the peptoid-based conjugates represent the first multivalent constructs designed to target the androgen receptor (AR). The oligomeric scaffold provides a versatile platform that can be employed to target AR activity. They concluded that multivalent e ethisterone ligands conjugated to peptoid oligomers can compete for binding to AR, and interfere with AR-mediated transcription. Linear and cyclic-designed peptoid oligomers exhibit potent anti-proliferative activity in therapy-resistant prostate cancer cells through competitive and non-competitive mechanisms, respectively.

Peptidomimetic P29 is a newly designed FGF2 (a member of the FGF family that belongs to cytokines) inhibitor that has possession high affinity with it. Li et al. (2016) showed that P29 suppressed the FGF2-induced proliferation of gastric cancer (GC) cells. It also inhibited the phosphorylation of FRS2, ERK1/2, and AKT triggered by FGF2 in GC. In addition, P29 blocked GC cell transformation from the G1/G0 phase to the S phase.

Doxorubicin (DOX) is a well-known anticancer drug, but it has limited cytostatic effects in therapeutic doses. Pallerla et al. (2017) find that conjugation of DOX with a peptidomimetic (Argaminonaphthylpropionic acid-Phe) that is highly specific for HER2-overexpressed cancer cell lines, has antiproliferative activity against HER2-positive cancer cells such as BT-474, SKBR-3 (human breast tumor cell lines), and Calu-3 (human lung cancer cell line). In a similar study, Banappagari et al. (2010) reported that HERP5 (Arg-βNaph-Phe) as peptidomimetic designed based on the HER2–Trastuzumab interaction model to inhibit HER2-mediated signaling possesses antiproliferative activities by binding to the extracellular region of the HER2 protein in BT-474 cell lines. Furthermore, Naik et al. (2018) designed a novel peptidomimetic and conjugated it with stearic acid at the N-terminal of the peptide (Stearic acid-Arg-(S)Anapa-Phe-OH). They showed that this conjugated peptidomimetic could have antiproliferative activity in lung cancer cell A549, and other cell types like Calu-3 and BT-474 that overexpressed HER2 by binding specifically to the HER2 extracellular domain, particularly to domain IV, and inhibits human epidermal growth factor receptor (EGFR) heterodimerization. Besides, Shanthi et al. (Kanthala et al. 2017) targeted EGFR heterodimerization by Cyclo(1,10)PpR (*R*) Anapa-FDDF-(*R*)-Anapa)R peptidomimetic and reduced proliferation in breast and lung cancer cell lines.

In a study that was conducted to design and develop anti-cancer agents, Donkor et al. (2020) synthesized four sulfonamide-based peptidomimetics as inhibitors of μ-calpain (calcium-activated cysteine proteinases) that incorporate (E)-1-(phenyl)-2-phenyldiazene and (E)-1-(phenyl)-2-phenylethene functionalities as the N-terminal capping groups of the inhibitors. Their in vitro analysis outcomes revealed that these peptidomimetic compounds displayed moderate to strength antiproliferative activity versus melanoma cell lines (A-375 and B-16F1) and PC-3 prostate cancer cells. RGD-based peptidomimetics also have anti-proliferative properties. S137 is a low-molecular-weight peptidomimetic of the ligand RGD that is most active in inhibiting ligand binding to the family of five integrins which contain the alpha v subunit. Shannon et al. (2004) reported that treatment of cultured human microvascular endothelial cells completely abolished their proliferation at concentrations above 5 μM, regardless of whether bFGF or VEGF was utilized as a mitogen.

The α-amanitin is a cyclic peptide of eight amino acids that inhibit cellular transcription by an effective blocking of RNA polymerase II. Due to high polarity and poor membrane permeability, α-amanitin exhibits micromolar cytotoxicity and low cellular uptake in most mammalian cells. To address this problem, Bodero et al. (2018) conjugate this cyclic peptide to RGD and isoDGR peptidomimetic, and evaluate their ability for tumor-targeting. The results of this experiment showed that designed conjugates have antiproliferative activity against cancer cell lines such as U87 (human glioblastoma cell line), A549, and MDA-MB-468.

L-744,832 (2(s)-[2(s)-[2(R)-amino-3-mercapto]-propylamino-3(s) methyl] pentyloxy-3-phenylpropionyl methionine-sulfone isopropyl ester) is a peptidomimetic inhibitor of protein farnesyltransferase. Farnesylation of the CAAX box in the COOH terminus of Ras protein by protein farnesyltransferase is an essential step in the functional activation of the Ras pathway and transformation (DeFeo et al. 1981; McKay et al. 1986). Sirotnak et al. (2000) reported that L-744,832 could be safely administered over a protracted period of time to mice at doses that were markedly inhibitory to the growth of TSU-PR1, DU-145 and PC-3 (human prostatic carcinoma cell lines) xenografted to NCR-nu1 (AT) mice and in combination with paclitaxel (PTX) was also well-tolerated and brought about some regression of the TSU-PR1 tumor by inhibition of Ras functionality. Furthermore, Tsubamoto et al. (2019) designed a guanidyl-based bivalent peptidomimetic to target K-Ras (as the most frequently mutated Ras isoform in human cancers) prenylation and showed its antiproliferative activity in T24 human bladder carcinoma cells.

Chauhan et al. (2014) designed a pyridyl peptidomimetic and evaluated its ability to inhibit proliferation. They find that designed peptidomimetics can diminish the proliferation of HepG2 human hepatocellular liver carcinoma cells by targeting c-KIT1. Wang et al. (2017) designed ERG inhibitory peptides and derived peptidomimetics and proved their antiproliferative activity by proteolytic degenerating of ERG in prostate cancer models. Levesque et al. (2015) used PACE4 inhibitors peptidomimetic analogs to inhibit the proliferation of prostate cancer cells. Xia et al. (2016) demonstrated that some peptidomimetic analogs of XK469 have antiproliferative activity via DNA topoisomerase II (topo II) inhibition on hepatoma H22. Dal Corso et al. (2015) also showed that RGD peptidomimetic–Paclitaxel conjugates are stranger inhibitors of proliferation of the human leukemic cell line CCRF-CEM cell line compared to free Paclitaxel.

## Peptiodomemtics against metastasis

It is common for patients with a first-time disease diagnosis to have disseminated cancer cells. Therefore, medicines may become ineffective in treating metastatic cancers that exclusively target the early stages of the disease (Malla et al. 2022). An alternative to antagonizing early events is to weaken the ability of already-migrated cells to survive and reproduce in their non-native environments. Such therapy would not only inhibit the growth of undetected tumor foci but could reduce the risk of metastasis. Wang et al. (2017) tested Retroinverso-ERG inhibitory peptides1 (RI-EIP1) for ERG-mediated metastasis in an animal model. They implanted TMPRSS2: ERG-positive VCaP (cell line of human prostate cancer) onto a fertilized chicken embryo’s upper chicken chorioallantoic membrane (CAM). After removing chicken lungs and measuring the number of metastatic VCaP cells, the results showed that RI-EIP1 treatment significantly reduced lung invasive cells from VCaP tumors, indicating the anti-metastatic effects of RI-EIPs (Wang et al. 2017).

In another study, Shukla et al. (2021), identified a peptidomimetic with unique characteristics (called PCS2D1.2) that specifically targets the cancer stem cells (CSC) subpopulation of lung cancer on residual cancer cells (non-CSCs). Unlike PCS2 peptoid, which did not exhibit anticancer activity, PCS2D1.2 shows high anti-cancer potency. According to the results of this investigation on the H358 cell line (lung cancer), PCS2D1.2 significantly inhibited cell metastasis by inhibiting known markers of CSC ALDH1A3, SOX2, and pectin. PCS2D1.2 specifically affects CSCs, while does not affect non-CSCs (Shukla et al. 2021).

The binding of amyloid precursor protein (APP) to death receptor 6 (DR6) eased the necrosis (necroptosis) pathway, and knocking down either APP or DR6 results in a considerable reduction of tumor cell (TC)-induced necroptosis and TCs transendothelial migration (Strilic et al. 2016). The connection between APP and DR6 could be a promising new target for anti-hematogenous metastatic treatments. However, finding DR6/APP interaction inhibitors with high binding affinity and appropriate pharmacokinetic properties in the blood system is extremely challenging. Wang et al. (2021) in their research, showed that polymer–peptidomimetic conjugate (PEG-tAHP-DRI) could significantly suppress hematogenous metastasis in serval different metastatic mouse models (B16F10, 4T1, CT26, and spontaneous lung metastasis of 4T1 orthotopic tumor model). This highlights PEG-tAHP-DRI as a DR6/APP inhibitor with promising potential for developing anti-hematogenous metastatic agents with long-acting and no apparent toxicity (Wang et al. 2021; Xu et al. 2015).

Angioputin-like protein 2 (ANGPTL2) is one of the critical factors in inflammatory carcinogenesis and tumor metastasis. ANGPTL2 has been shown to cause osteosarcoma metastasis in prior studies (Odagiri et al. 2014). Due to the antitumor activity shown for GDC-0152, a small peptidomimetic molecular antagonist of apoptosis inhibitor proteins, Yang et al. (2015) studied the interaction between ANGPTL2 and GDC-0152. ANGPTL2-induced osteosarcoma was examined in this study for its effect on malignant progression. They exposed human osteosarcoma cell line SaOS2 cells to recombinant human ANGPTL2 after pre-treating or not treating the cells with GDC-0152. As a result of treatment with ANGPTL2, SaOS2 cells’ growth and migration increased, and their apoptosis was reduced. Interestingly, GDC-0152 attenuated the malignant progression of osteosarcoma promoted by ANGPTL2 via the PI3K/AKT signaling pathway (Yang et al. 2015).

**Fig. 4:**
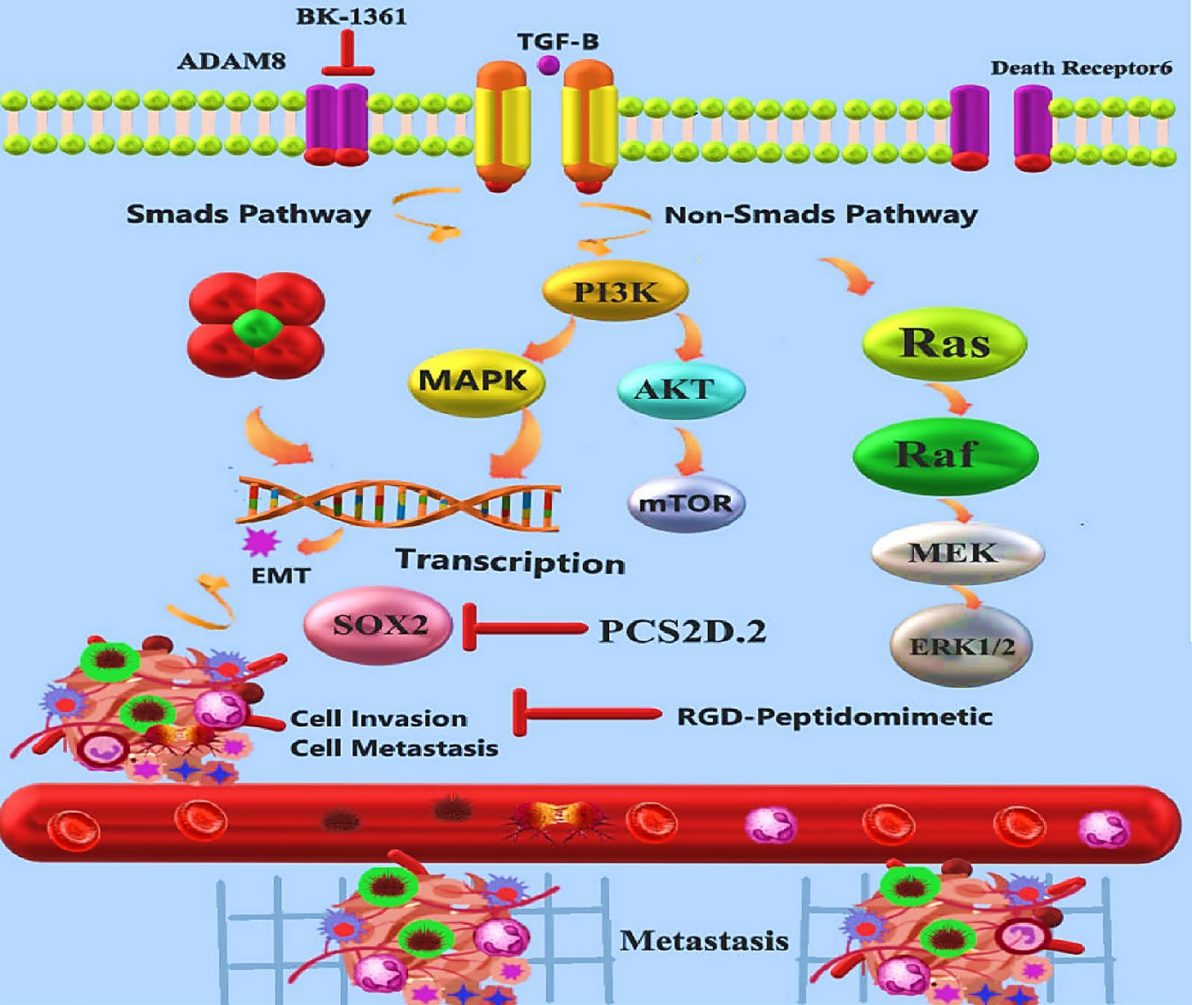
Inhibition of cancer cells metastasis potency using peptidomimetics

Other studies have been done on peptidomimetics in treating pancreatic ductal adenocarcinoma (PDAC). According to Schlomann et al. (2015) experiments, a disintegrin and metalloproteinase 8 (ADAM8) overexpression allows PDAC cells to migrate and invade. This overexpression is due to higher matrix metalloprotease (MMP) activity and extracellular signal-regulated kinase (ERK1/2) activation. ADAM8 interacts with integrin b1 at the cell surface and requires multimerization for biological function. BK-1361, a peptidomimetic ADAM8 inhibitor created using disintegrin domain structural modeling, suppresses ADAM8 multimerization. BK-1361 inhibits ADAM8 function in PDAC cells, decreasing invasiveness and ERK1/2 and MMP activation (Fig. 4). In a KrasG12D-driven animal model of PDAC, BK-1361 treatment reduced tumor burden and metastasis of implanted pancreatic tumor cells and improved clinical symptoms and survival rates (Schlomann et al. 2015).

Shannon et al. (2004), analyze the efficacy of 2 structurally similar RGD peptidomimetic agents in two different models of metastasis by characterizing them in vitro activities in detail. In the first model, there is a spontaneous model that encompasses all steps of the metastatic process, employing the human 435/HAL line, which is an aggressive variant of the MDA-MB-435 line that contains the GFP gene. The second model involves injecting B16-F10 murine melanoma cells into the veins of syngeneic recipients. The involvement of integrins in these activities has been studied by previous studies. The results indicated the effectiveness of the designed peptidomimetics in inhibiting metastasis.

## New opportunity of peptidomimetic for cancer drug resistance

Multidrug resistance (MDR), the phenomenon when cancer becomes resistant to structurally and functionally diverse classes of drugs, often contributes to the failure of chemotherapy to treat various types of cancer (Wang et al. 2022). The increase in active efflux of a drug by ATP-binding cassette (ABC) transporters appears to be a major contributor to acquired resistance, among the various mechanisms associated with MDR such as the decline in drug uptake, cell cycle progression, drug metabolism, augmented DNA repair, and decreased apoptosis. P-glycoprotein (P-gp/ABCB1/MDR1) is the most common ABC transporter that causes MDR in the superfamily of ABC transporters (Taskar et al. 2022). Over the past 30 years, there have been three generations of P-gp modulators developed as a result of research on the reversal of MDR; due to a variety of reasons, however, these modulators failed to show effectiveness in clinical trials. Part of the reason for the failure was that the medication lacked bioavailability at tumor sites and caused nonspecific toxicities (Abdelaal and Haffez 2022).

In clinical trials of P-gp modulators, concerns have also been raised about selecting appropriate patients. For the selection of patients, it is important to consider the causal factor for MDR, including the ABC transporter that was developed specifically to modulate. As part of the patient selection process, single nucleotide polymorphisms (SNPs) should also be evaluated (Anglicheau et al. 2003). Inflammatory diseases can also significantly influence treatment outcomes in patients. Nevertheless, these efflux transporters play a crucial role in the development of MDR and merit further study to ensure the best possible clinical outcome. It is crucial in oncology settings to treat cancers resistant to currently available P-gp inhibitors to develop new classes of compounds devoid of the limitations associated with previously disclosed compounds. Therefore, reversal compounds with high affinity and selectivity for P-gp are being developed to reverse MDR (Altinoz et al. 2022).

Ma et al. (2012), discovered that lysine-peptidomimetic (Lys-P) is a robust synthetic peptidomimetic that reverses P-glycoprotein (P-gp) mediated MDR via a novel mechanism involving non-competitive suppression of P-gp ATPase activity and no substantial change in P-gp expression. They designed an experiment on P-gp-mediated MDR in human embryonic kidney 293 (HEK293) cells, for investigation of the Lys-P on Dox cytotoxicity and intracellular accumulation of another P-gp substrate, rhodamine 123. Also, more analysis was conducted in colorectal adenocarcinoma cells (Caco-2) to determine the effect of Lys-P on P-gp expression. Lys-P restored Dox cytotoxicity in resistant MDR1-transfected HEK293 and MCF-7 TX400 cells while not affecting their parental cells. In HEK293 MDR1 cells, it also significantly boosted intracellular rhodamine 123 accumulation (21-fold). Further mechanistic studies revealed that Lys-P eliminated P-gp-mediated Dox efflux in the Caco-2 cell monolayer model due to uncompetitive inhibition of P-gp ATPase without changing P-gp expression. Based on this research, Lys-P was a promising lead compound for further development into selective and efficient MDR reversing agents for use with P-gp substrates in cancer chemotherapy (Ma et al. 2012).

A newly synthesized thiazole-valine peptidomimetic, TTT-28, was designed to reverse ATP-binding cassette B1 (ABCB1) mediated MDR in vitro and in vivo by Wang et al. (2017). ABCB1 overexpressing cells were treated with TTT-28 to reverse the ABCB1-mediated MDR and increase [3H]-paclitaxel accumulation by selectively blocking ABCB1 efflux. The animal study found that TTT-28 significantly increased intratumoral paclitaxel concentrations and promoted apoptosis, inhibiting the growth of ABCB1 overexpressing tumors. TTT-28 did not cause the same toxicity as paclitaxel in mice (cardiotoxicity and myelosuppression). In addition to facilitating the design and synthesis of a new generation of ABCB1 inhibitors, the discovery and characterization of this new thiazole-valine peptidomimetic may provide valuable information regarding multidrug resistance and combination chemotherapy. Also, co-administering MDR-ABCB1 inhibitors to overcome the resistance of a widely used chemotherapeutic that is FDA-approved could be a helpful strategy for adjuvant chemotherapy (Wang et al. 2017).

To date, several experiments have been performed to examination of peptidomimetics therapeutics effects on macrophages, due to the role of these immune cells on chemotherapy and drug resistance (Xiao et al. 2021; Ruffell and Coussens 2015). Proprotein convertase (PC) is a member of the subtilisin/Kexin family that cleaves proproteins through limited proteolysis and converts them into proteins or peptides (Jaaks and Bernasconi 2017; Rose et al. 2020; Day and Salzet 2002). Rose et al. (2020) examined the ability of proprotein convertase inhibitors to macrophage reactivation. In these experiments, macrophages were treated with a PC inhibitor (C35H55N15O4) peptidomimetic that inhibits proprotein convertases such as furin, PC1/3, PC4, PACE4, and PC5/6. Compared with temozolomide, a drug used to treat gliomas, PC inhibitor (C35H55N15O4) anti-glioma activity is more effective at reducing cancer cell density. Furthermore, the PC inhibitor (C35H55N15O4) recruited macrophages into the tumor, such as CCL6 and PRDX5, by secreting several immune factors with anti-tumor effects. These results demonstrated that the protein converter inhibitor could be used as a glioma drug and an anti-tumoral macrophage reactivation agent. In treating gliomas, this approach can be used in combination with chemotherapy to increase its effectiveness (Rose et al. 2020).

**Fig. 5:**
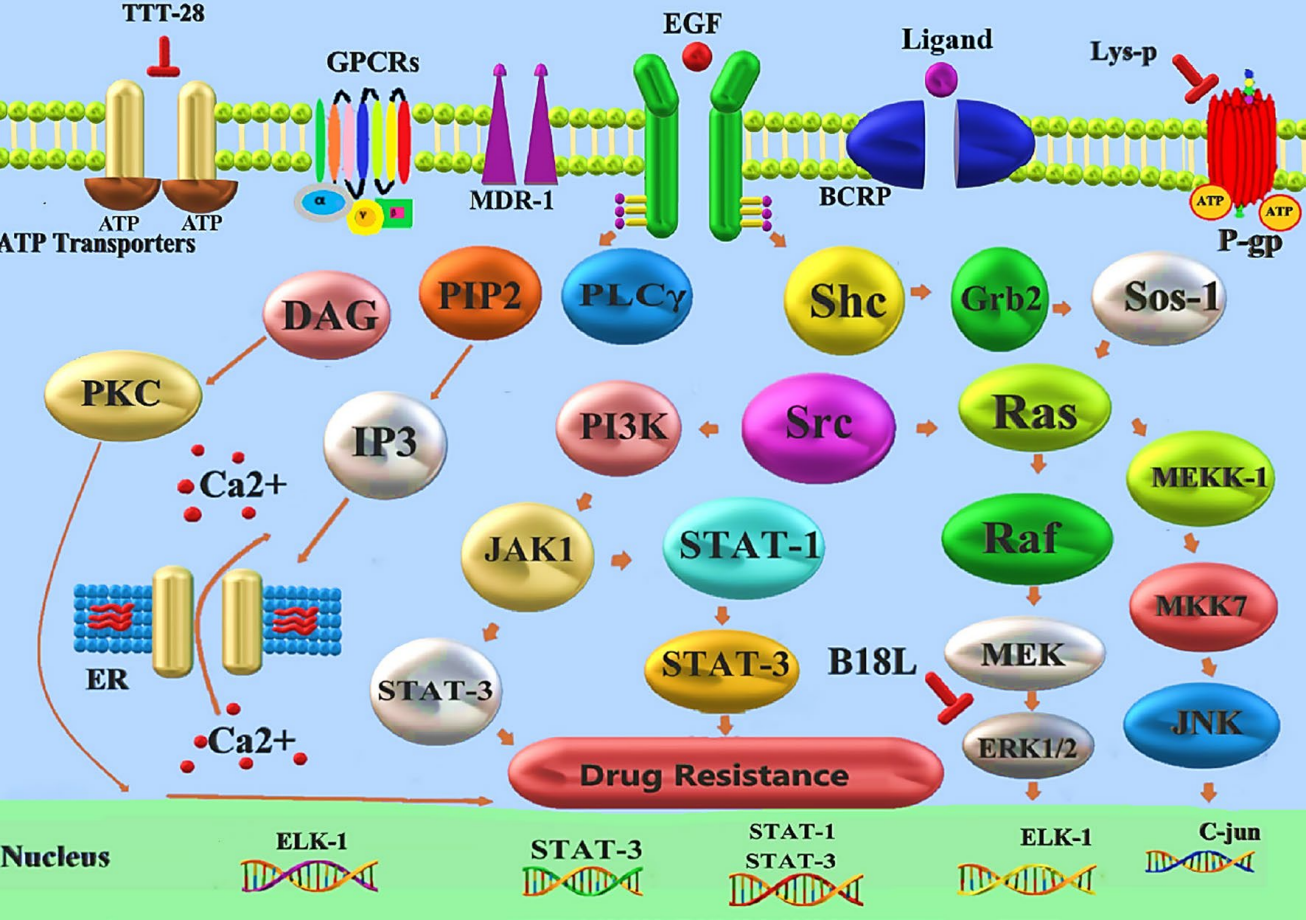
Targeting drug resistance pathways by utilizing peptidomimetics

Several studies revealed that bone marrow stromal antigen-2 (BST-2) is overexpressed in breast cancer cells, leading to increased adhesion and invasion of these cells. The level of BST-2 in breast cancer tumors is higher than cancer markers, but so far, no effective method has been developed to target BST-2. Thus, targeting BST-2 may be a versatile option for the treatment of breast cancer patients (Mahauad-Fernandez and Okeoma 2018). As the backbone of their cytotoxic anti-cancer agent, Yuan Lyu et al. (2020) used the BST-2 anti-adhesion peptide B18, a second-generation anti-adhesion peptide against BST-2. They designed five peptidomimetics based on B18. One of them, B18L, an amphiphilic cationic α-helix peptidomimetic, was chosen for development as the lead drug because of its superior anti-cancer activity against drug-resistant and drug-sensitive cancer cells with minimal toxicity to normal cells. Functionally, B18L therapeutics effects appear following binds to BST-2. B18L works by coordinating membrane functions, mitochondrial function, and signaling (Fig. 5). Studies on B18L have shown that this peptidomimetic decreases Src and Erk1/2, enhances Bcl2 pro-apoptotic proteins, and triggers caspase bed processing in breast cancer (Lyu et al. 2020).

## Integration of peptidomimetic and nanotechnology

The highly effective nature of nanoparticles (NPs) in overriding the body’s natural defense system and its vascular barriers has led to a wide range of applications in medicine and pharmacy. A few of the applications are drug delivery, vaccine, biological labeling, examination and test of the DNA, separating of the molecules and cells, imaging in medicine usages, and phagokinetic studies. Polymers (synthetic macromolecules) can be extremely flexible in the design of nanoparticles, allowing certain NPs to have precise drug solubility, sizes, and degradation characteristics to satisfy specific applications (Wang et al. 2022).

Nanocarrier-mediated chemotherapeutic administration can improve the therapeutic potency of anti-cancer agents. Ligand-targeted drug delivery can be utilized to deliver chemotherapeutic drugs to cancer cells in a selective and precise manner. In a study conducted by Naik et al. (2021), they created a peptidomimetic conjugate (SA-5)-tagged Dox incorporated liposome (LP) formulation (SA-5-Dox-LP) to assess the targeted delivery potential of SA-5 in HER2-overexpressed non-small-cell lung cancer (NSCLC) and breast cancer cell lines. The antiproliferative activity of targeted SA-5-Dox-LP was superior to non-functionalized Dox liposomes in cell viability experiments (Dox-LP). When HER2-targeted liposomes were delivered to cancer cells, they displayed preferential cellular uptake compared to non-functionalized liposomes. Dox was released slowly from the formulations for 24 h, according to in vitro drug release experiments. There was no change in Dox release between the Dox-LP and SA-5-Dox-LP formulations. Studies in mice using a lung tumor model of NSCLC showed that SA-5-Dox-LP significantly reduced lung tumors compared with vehicle controls (Naik et al. 2021).

Given the importance of the role of angiogenesis in cancer progression, anti-angiogenic therapy offers a promising therapeutic approach, alone or with conventional treatment protocols. PTX is a potent cytotoxic drug exhibiting anti-angiogenic effects at low doses. However, its use is limited by severe side effects to its full potential. Eldar-Boock et al. (2011) developed a conjugate platform for PTX, a polymer (PGA)-PTX-E-[c(RGDfK)2]. The designed platform passively targets the tumor tissue and enhances the permeability and retention of PTX in the tumor environment. The cyclic RGD peptidomimetic significantly enhanced these effects. They showed that PGA is enzymatically-degradable, leading to PTX release under lysosomal acidic pH. PGA-PTX-E-[c (RGDfK)2] inhibited the growth of avb3-expressing endothelial cells and several cancer cells. Furthermore, PGA-PTX-E-[c(RGDfK)2] blocked endothelial cells’ migration towards vascular endothelial growth factor, and capillary-like tube formation, and inhibited endothelial cells’ attachment to fibrinogen in the 4T1 breast tumor model. Orthotopic studies in mice demonstrated preferential tumor accumulation of the RGD-bearing conjugate, leading to enhanced anti-tumor efficacy and a marked decrease in toxicity compared with free PTX-treated mice (Eldar-Boock et al. 2011).

PTX can also be conjugated to a dendritic polyglycerol sulfate (dPGS) nanocarrier, which can cross the Blood–Brain Barrier (BBB), bind to P/L-selectins, and accumulate selectively in brain tumors. Because P-selectin is expressed in tumor endothelium and glioblastoma cells, dPGS provides dual targeting properties. By inducing Fas and Fas-L, dPGS-PTX in combination with the peptidomimetic anti-angiogenic protein thrombospondin-1 (TSP-1 PM) was able to cause a powerful synergistic anticancer impact on cerebral glioblastoma in people and animals. Compared to free PTX or temozolomide, the designed delivery system (dPGS-PTX-TSP-1) has shown fewer side effects (Ferber et al. 2017).

In the melanoma model B16F10, RNAi-mediated expression of STAT3 in tumor tissue reduces tumor development significantly. Ehexige et al., tested peptidomimetic-based lipid nanoparticles (LNPs) for siRNA delivery in a melanoma B16F10 mouse model. The innovative formulation (DoCh) delivered siRNA to tumor tissue preferentially when given systemically. Sequential intravenous injections of STAT3 siRNA resulted in profound suppression of STAT3 expression in tumor tissue, resulting in considerable downregulation of programmed death ligand‐1 (PD-L1) and significant tumor growth inhibition via inactivation of the tumor immune checkpoint. Furthermore, the major organs were not damaged by DoCh-mediated siRNA delivery (Ehexige et al. 2020). Moreover, another experiment showed that cholesterol-based lipid nanoparticles functionalized with cationic peptidomimetic delivered siRNA against polo-like kinase 1 (Plk1). This platform effectively suppressed the gene expression in cancer cells, resulting in significant morphological changes in the nucleus of the cancer cells (Ehexige et al. 2019).

Researchers have created new peptidomimetic nanoassemblies that include a fluorescent semiconductor nucleus (AgInS2, AIS), cysteine-modified carboxymethylcellulose (called a thiomer, CMC Cys), mitochondrial target peptides (KLA) to form macro magnetic particle to treat malignant brain tumors. Compared to doxorubicin, peptidomimetic nanohybrids have been demonstrated to be more fatal against brain cancer cells (U-87 MG). Significantly, these peptidomimetic nanohybrids had a “protective impact” on healthy cells while still killing malignant brain cells at a significant rate (Carvalho et al. 2021).

PEGylated chitosan-based nanoparticles are appealing platforms for siRNA cocktails delivery to tumors. Targeting two important energy fuel carriers for cancer cells, lactate transporter MCT1 and glutamine transporter ASCT2, with non-covalent PEGylation of chitosan-based nanoparticles loaded with siRNA, can lead to strong antitumor effects. In mice, peri-tumoral and systemic injections of siRNA and selected target nanoparticle components, along with RGD peptidomimetic (RGDp), significantly reduced tumor growth (Corbet et al. 2016).

## Conclusion

Pharmacology and drug design advances have raised expectancies for cures and improvement of cancer patient’s health status. Peptides are among the most popular drugs available to target cancer cells because of their small size and ease of production. But disadvantages such as a high rate of clearance, low penetration potency to the desired tissue, immunogenicity, toxicity, and low affinity to the target have limited their use. Peptidomimetics are a worthy alternative to peptides and a viable option against cancerous cells. However, disadvantages like low biocompatibility and complicated production have hampered the development of peptidomimetics. In recent years, tremendous progress has been made in the field of synthetic production of peptides with various modifications, like cyclic peptides. Besides, the advances in computational biology, structural biology, and peptide engineering have solved some of the limitations and facilitated the use of peptidomimetics in cancer investigations. Low-cost and high-speed computational approaches have facilitated the production of peptides with modified structures. Things like molecular docking, simulation of molecular dynamics, peptide quantitative structure–activity relationships (peptide QSAR), and the possibility of predicting the 3D structure of the peptide complex with its target, are among the advances in the computational design of chemically modified peptides.

## Data Availability

Not applicable.

## Abbreviations

Amba: Amidinobenzylamide
APP: Amyloid precursor protein
AR: Androgen receptor
ABC: ATP-binding cassette
ABCB1: ATP-binding cassette B1
BBB: Blood–brain barrier
BST-2: Bone marrow stromal antigen-2
CSC: Cancer stem cells
CAM: Chorioallantoic membrane
Caco-2: Colorectal adenocarcinoma cells
CT: Computed tomography
DR6: Death receptor 6
dPGS: Dendritic polyglycerol sulfate
DKP: Diketopiperazine
DOX: Doxorubicin
EGFR: Epidermal growth factor receptor
ERK1/2: Extracellular signal-regulated kinase
GC: Gastric cancer
GRAB: Group replacement assisted binding
HUVEC: Human umbilical vein endothelial cells
IAC: Integrin antagonist
IFNγ: Interferon-γ
LNPs: Lipid nanoparticles
LP: Liposome
Lys-P: Lysine-peptidomimetic
MRI: Magnetic resonance imaging
MMP: Matrix metalloprotease
MVD: Microvessel density
MDR: Multidrug resistance
ML: Multi-Leu
NPs: Nanoparticles
NK: Natural killer
NSCLC: Non-small-cell lung cancer
PTX: Paclitaxel
PDAC: Pancreatic ductal adenocarcinoma
P-gp: P-glycoprotein
Plk1: Polo-like kinase 1
PET: Positron emission tomography
PD-L1: Programmed death ligand‐1
PC: Proprotein convertase
PPIs: Protein–protein interactions
QDs: Quantum dots
RI-EIP1: Retroinverso-ERG inhibitory peptides1
Arg-Gly-Asp: RGD
RGDp: RGD peptidomimetic
SNPs: Single nucleotide polymorphisms
SPECT: Single-photon emission computed tomography
STAT3: Signal transducer and activator of transcription 3
topo II: Topoisomerase II
TC: Tumor cell
